# Cognitive Frailty: An Update

**DOI:** 10.3389/fpsyg.2021.813398

**Published:** 2021-12-16

**Authors:** David Facal, Clara Burgo, Carlos Spuch, Pedro Gaspar, María Campos-Magdaleno

**Affiliations:** ^1^Department of Developmental and Educational Psychology, University of Santiago de Compostela, Santiago de Compostela, Spain; ^2^Galicia Sur Health Research Institute, Vigo, Spain; ^3^UNICES, Universidade da Maia, Maia, Portugal

**Keywords:** frailty, subjective cognitive decline, cognitive reserve, biomarkers, physical activity, dual-task intervention, multicomponent intervention programs, mild cognitive impairment

## Abstract

This review article provides an update of the empirical research on cognitive fragility conducted in the last four years. The studies retrieved were classified in four different categories. The first category includes articles relating cognitive frailty to cognitive reserve and which continue to highlight the importance of educational level. The second category includes recent research on cognitive fragility biomarkers, involving neuroimaging, metabolism and, in a novel way, microbiota. The third category includes research on how cognitive frailty is related to motor development and physical functioning, exploring e.g. the use of technology to study motor markers of cognitive frailty. Finally, in the fourth category, research clarifying the difference between reversible frailty and potentially reversible cognitive frailty has led to new interventions aimed at reducing cognitive frailty and preventing negative health outcomes. Interventions based on physical activity and multicomponent interventions are particularly emphasized. In addition, recent research explores the long-term effects of dual interventions in older adults living in nursing homes. In summary, research on cognitive frailty has increased in recent years, and applied aspects have gained importance.

## Introduction

The International Academy on Nutrition and Aging (I.A.N.A) and International Association of Gerontology and Geriatrics (I.A.G.G) consensus group has defined cognitive frailty as the simultaneous presence of physical frailty (PF) and mild cognitive impairment (MCI) in the absence of dementia or other pre-existing brain disorders (Kelaiditi et al., 2013). Facal et al. (2019) conducted a systematic review with the aim of analyzing the definition of the term “cognitive frailty” in the empirical literature published up to August 2017. The authors concluded that a more comprehensive definition of the potential relationships between PF and MCI was needed. They also indicated some limitations regarding the scarcity of specific markers of cognitive reserve and motor impairment and the lack of interventions studies.

Since then, research on cognitive frailty has increased exponentially. This review study aims to provide an update of research on the topic published in the empirical literature. Two independent authors conducted an empirical literature search in Medline, Web of Science, PsycINFO and Cochrane databases from September 2017 to December 2020, with the term “cognitive frailty”. All original empirical studies in English, Spanish or Portuguese that explicitly used the term “cognitive frailty” were included. In total, 64 records were obtained from Medline, 73 from Web of Science, 9 from PsycINFO and 15 from Cochrane. After removal of duplicates, 80 articles were considered. Two independent authors reviewed the title, abstract and keywords and evaluated their suitability for inclusion. Any conflicts were discussed until consensus was reached. According to the structure proposed by Facal et al. (2019), only articles that explicitly measured cognitive reserve, biological markers, motor capacity or that involved intervention studies were included. In their 2019 systematic review, Facal et al., analyzed all scientific research including the term “cognitive frailty” up to August 2017, and extracted these four thematic areas as the most relevant in the study of cognitive frailty as an applied concept in psychogerontology. As an update, in this mini-review we have decided to continue with this structure, and also to incorporate it as an inclusion criteria in order to maximize its potential for explanation. Finally, 4 articles concerning the relationships between cognitive frailty and cognitive reserve, 6 articles on the associations between cognitive frailty and biomarkers, 10 articles about motor signs and 8 articles analyzing the effects of interventions against cognitive frailty were selected (see Supplementary Material).

## Relationship Between Cognitive Frailty and Cognitive Reserve

Although the I.A.N.A-I.A.G.G definition indicates that cognitive frailty is characterized by reduced cognitive reserve, to date educational level has been the only proxy for this measure that has been systematically included in studies on cognitive frailty (Facal et al., 2019). We found that this trend continues in the most recent literature. Niederstrasser et al. (2019) detected a protective effect against early development of frailty and for frailty progression in individuals with any type of formal education, relative to individuals with no educational qualifications. Ruan et al. (2020) observed lower rates of physical and cognitive frailty in participants with an intermediate educational level (6–12 years) than in participants with a lower educational level. Similarly, Gallucci et al. (2020) reported that education seems to be a protective factor in the incidence of frailty, with more years of education associated with robust or pre-frailty and low education associated with frailty. Wongtrakulruang et al. (2020) associated low education level (primary school or less) a with higher risk of MCI and PF/pre-frailty.

Recent evidence reinforces the role of low educational level in the early stages of life as a strong, non-modifiable risk of cognitive frailty (Niederstrasser et al., 2019), highlighting the relationships between low wealth, low educational attainment and negative health outcomes, especially at the end of the lifespan, when older adults are more vulnerable to stressors. However, the relationship between cognitive frailty and cognitive reserve remains to be well established, by including not only measures related to years of formal education but also to proxies for work complexity or intellectually active lifestyles.

## Role of Biomarkers

Identification of biomarkers of CF is difficult as the syndrome is multidimensional. In the context of neuroimaging evidence, several recent papers have addressed the structure of certain areas and the damage caused by cerebrovascular diseases. Sugimoto et al. (2019) describe the relationship between inflammatory markers as a risk factor for white matter hyperintensity (WMH). By examining hyperintensity in T2-weighted and fluid-attenuated inversion recovery (FLAIR) images, these researchers demonstrate increased volumes of WMH in CF and prefrail (PF) participants. At the structural level, Wan et al. (2020) describe a significant reduction in five subcortical nuclei (bilateral thalami, left caudate, right pallidum, accumbens area and the bilateral thalami). The data reported suggest that CF is associated with loss of structure of the thalamus and hippocampus and changes in WMH, and that possible volumetric biomarkers in these areas could thus potentially act as biomarkers of CF and its progression.

Consideration of the microbiota is a new aspect in the development of age-related biomarkers. Changes in the microbiota during aging are increasingly being studied, and it has been suggested that potentially important changes occur during the development of CF. In a recent study, He et al. (2020) demonstrated that CF patients have elevated levels of trimethylamine N-oxide (TMAO), a stable metabolite of the intestinal microbiota.

At the metabolic level, plasma biomarkers that are easily identifiable in a routine blood test are being studied. After longitudinal analysis of a population of 7,769 individuals included in the Doetinchem Cohort Study, Rietman et al. (2019) found that none of the following parameters were predictive of CF: high-density lipoprotein (HDL) cholesterol, triglycerides, alanine aminotransferase (ALT), gamma-glutamyltransferase (GGT), C-reactive protein (CRP), albumin, uric acid, cystatin C or creatinine. Royal and Plamen (2019) found that insulin-like growth factor 1 (IGF-1) and IGF-binding protein 2 (IGFBP2) were implicated in CF and potentially in the cause of age-related cognitive decline and physical frailty.

Regarding metabolism, recent research has examined the importance of lipids both in cell structure and at the nutritional level. Considering that lipids are a major component (70%) of the composition of the human brain, variations in these compounds could potentially be used as biomarkers of cognitive problems. In recent research, Sargent et al. (2020) found that low levels of vitamin E alpha tocopherol, omega-6 and 3 and albumin were associated with CF. In addition, these researchers observed a second pattern of association characterized by a low level of trans fats, as indicated by measuring low and high density lipoproteins (LDL and HDL).

## Relationship With Motor Signs of Aging

Considering the importance of motoric aspects in the interplay between cognitive performance, cognitive impairment and PF, different studies on cognitive frailty have focused on motor decline and gait variables. Although Facal et al. (2019) pointed out that motor decline and gait variables were not systematically included in protocols for assessing cognitive frailty, more recent studies frequently include these aspects. For example, Armstrong et al. (2019) associated cognitive performance measured by a computerized Stroop with physical functioning in a randomized sample of 607 old adults in the Baltimore Experience Corps Trial. Slower initial performance in the computerized test, but not lower learning rate, was associated in this study with poorer performance in the short physical performance battery (SPPB). Simpler physical performance assessment tools such as the Timed Up and Go (TUG) test are also good indicators of cognitive frailty. A decrease in functional mobility measured with this test has been shown to be a significant predictor of both prevalence (Kim et al., 2019) and incidence of cognitive frailty (Rivan et al., 2020). In the latter study, a one-unit increase in TUG was found to significantly increase the risk of developing cognitive frailty in an older population. Wanaratna et al. (2019) reported a high prevalence of frailty and pre-frailty in community-dwelling older patients with osteoarthritis of the knee, and severe symptoms of osteoarthritis measured by Oxford knee score were also associated with cognitive frailty.

The incidence of falls in frail old adults is high, and the negative consequences of falls increase with age. Recent studies associate cognitive frailty with a higher risk of falls. Tsutsumimoto et al. (2018) reported a higher risk of falls and also an increase in the fracture risk after falling in cognitively frail old adults than in old adults with normal cognition. According to the findings of the study, cognitive frailty is associated with a greater risk for fall-related fractures than cognitive impairment or PF alone. Zhao et al. (2020) reported that the relationship between cognitive frailty and falls may be mediated by engagement in activity, considered as a lifestyle factor which decreases the risk of falling. In this regard, risk of falling may lead to reduced physical activity, but also to reduced engagement in social activities and increased social isolation, which could lead to further cognitive and functional impairment.

Adequate assessment of motor performance is important in the context of cognitive frailty. Common approaches for assessing cognitive frailty use tools with a limited capacity to track changes over time and that may not be suitable for older adults living in remote, rural areas. Wearable sensors have thus been proposed as a possible means of measuring daily activity, as they are practical and reproducible. Recent studies have shown the feasibility and effectiveness of using remote physical activity and sleep monitoring recorded via a pendant sensor worn on the chest to identify old adults with cognitive frailty (Razjouyan et al., 2020), and using remote physical activity monitoring to identify pre-frail old adults (Razjouyan et al., 2018). Zhou et al. (2018) went a step further and evaluated a wearable platform to demonstrate the feasibility and efficacy of detecting cognitive impairment via an ankle-worn sensor in a series of interactive, instrumented trail-making tasks. The authors used trail-making tasks to quantify motor planning errors, by analyzing patterns of actual and optimal ankle velocity. The authors suggest that this procedure may be a substitute for dual-tasking walking tests when gait assessment is not possible.

## Cognitive Frailty as a Reversible Condition and Preventive Interventions

Recent research studies have attempted to differentiate between reversible and potentially reversible cognitive frailty. Cognitive frailty is considered reversible in the combined presence of physical pre-frailty (PF) and pre-MCI subjective cognitive decline (SCD), and potentially reversible in the combined presence of physical PF and MCI (Ruan et al., 2020). Reversible cognitive frailty is the ideal target to prevent asymptomatic, pre-clinical cognitive impairment. For this reason, and because it would be a central part of this pattern of reversible frailty, SCD has been the subject of recent research in the study of cognitive frailty. Hsieh et al. (2018) reported that old adults with SCD were more likely to be identified as pre-frail or frail than old adults with normal cognitive aging, regardless of potential confounding factors such as age, gender, education level, comorbidity, nutritional status, kidney function and biochemical-related factors. Okura et al. (2019) found that the impact of self-reported mobility decline (SR-MD) and self-reported cognitive decline (SR-CD) on adverse health outcomes depended on the moderating role of age and sex. For community-dwelling old men, SR-MD and non-SR-CD significantly predicted adverse health outcomes, with earlier negative outcomes than in non-SR-MD and SR-CD. For women, similar results were observed for respectively non-SR-MD and SR-CD, relative to SR-MD and non-SR-CD. Ruan et al. (2020) also observed gender differences, concluding that females with higher levels of education had a significantly increased risk of reversible cognitive frailty. Interestingly, in this study SCD was positively associated with physical pre-frailty but negatively associated with PF.

Although intervention studies in cognitive frailty are recent and relatively scarce, there is a growing consensus that interventions can be effective and beneficial in reducing cognitive frailty and/or preventing negative health outcomes. Interventions focused on physical activity and multicomponent interventions are highlighted. Regarding interventions based on physical activity, moderate-to-vigorous physical activity has been found to have a positive effect on cognitive frailty. Liu et al. (2018) reported that a 24-month structured programme of moderate-intensity physical activity was associated with a lower probability of worsening cognitive frailty. Similarly, an eHealth physical activity programme conducted over 12 weeks, promoting exercise in the form of brisk walking, was shown to reduce frailty and had a positive effect on mobility, improving cognitive function, walking time, step count, brisk walking time, peak cadence and moderate-to-vigorous activity time after the intervention (Kwan et al., 2020). Yoon et al. (2018) tested the effects of a high-speed resistance training programme conducted over 16 weeks in cognitively frail community-living older adults. The results showed that the exercise involved in the intervention improved cognitive function, physical function and muscle strength.

Adding cognitive intervention to physical exercise and due to the mutual influence between physical and cognitive decline, multicomponent programmes appear to be important for preventing and reducing cognitive frailty. Gallucci et al. (2020) found that frailty status was likely to improve by more than three times in participants of a 12-months structured programme including two/days week physical activity and a bimonthly group reading activity than in a control group who decided not to engage in the programme. Romera-Liebana et al. (2018) found that a multifaceted intervention including exercise training, intake of hyperproteic nutritional shakes, memory training and medication review was effective in reversing frailty 3 and 18 months after the intervention, improving mobility, balance, stretching, muscle strength and all dimensions of a neuropsychological battery and also reducing the number of medication prescriptions.

Finally, recent research has explored dual-task interventions in older adults living in long-term nursing homes. The ability to perform dual or multiple tasks decreases with age, particularly in the presence of cognitive impairment. According to Rezola-Pardo et al. (2019), dual-task interventions require greater cognitive and motor resources, are more complex in terms of control and coordination demands, and they may prevent or reverse frailty in older adults living in long-term nursing homes by improving cognitive function, gait and dual-task performance. These researchers compared a dual-task training intervention and a multicomponent exercise programme. In the dual-task training, the exercise component, based on the same physical exercises done by the groups undertaking the multicomponent exercise programme, was implemented with simultaneous progressive cognitive training. Cognitive exercises, including attentional, executive and semantic memory tasks, were individually tailored by adapting the difficulty for different cognitive domains for each participant. Both programmes were effective in improving gait and maintaining cognitive performance, and frailty status tended to improve. Nevertheless, the addition of simultaneous cognitive training did not provide additional benefits.

## Discussion

It has been possible to verify some important advances in research on cognitive frailty in the scientific literature. Links between cognitive frailty and years of education continue to highlight the relationships between low wealth, low educational attainment and negative health events (Gallucci et al., 2020; Ruan et al., 2020; Wongtrakulruang et al., 2020), and the mediational role of active engagement in the relationship between cognitive frailty and falls (Zhao et al., 2020) appears as an emerging research topic. It is desirable that research on cognitive frailty progressively incorporates other relevant proxies of cognitive reserve, such as leisure activity and work complexity, as well as global measures of the cognitive reserve construct.

The articles included in this review also support the relationship between cognitive development and motor development in cognitive frail older adults. The findings reported by Kim et al. (2019) and Rivan et al. (2020) highlight the importance of simple mobility tests such as TUG in the context of cognitive frailty. Decline in physical function can lead to reduced physical activity and socialization, which could lead to further functional decline and cognitive frailty. According to these authors, the interventions required to improve TUG performance may also be effective in preventing cognitive frailty and subsequent falls. Innovative devices such as portable sensors are also proposed as a possible alternative means of measuring daily activity in these populations.

Recent research also shows that a combination of clinical, inflammatory and neuroimaging markers could be included in a panel of clinically useful biomarkers for CF and the possibility of intervening at the nutritional and/or psychosocial level to reduce incident dementia. Lifestyle aspects such as physical activity and nutrition have been considered in preventive intervention measures aimed at mitigating physical and cognitive decline.

Finally, recent research provides the first evidence supporting the effectiveness of interventions designed to reduce cognitive frailty and prevent related negative health outcomes. These include interventions focused on physical activity and also multicomponent interventions. Interestingly, a dual task programme was effective in improving gait and maintaining cognitive function, but it did not produce better results than an equivalent, exercise-only programme (Rezola-Pardo et al., 2019). This result stress the protective role of physical exercise. It also suggest us that dual-task interventions must be tailored on the basis of physical performance under single-task conditions but also on the cognitive abilities and preferences of the participants in order to maximize their potential efficacy.

According to the promising results in intervention studies, reversibility remains an important aspect of research on CF, and this importance is expected to increase with successive research on preventive interventions and the role of SCD in the study of cognitive frailty. The development of clinical, inflammatory and neuroimaging markers of CF would also help in differentiating between reversible and potentially reversible CF and in designing more precise preventive studies.

## Author Contributions

All authors listed have made a substantial, direct, and intellectual contribution to the work and approved it for publication.

## Funding

This work was financially supported through FEDER founds (A way to make Europe) by the Spanish AEI (doi: 10.13039/501100011033; Refs. PID2020-114521RB-C21 and PSI2017-89389-C2-1-R) and by the Galician Government (Consellería de Cultura, Educación e Ordenación Universitaria; GI-1807-USC: Ref. ED431C 2021/04). CB was supported by a Research Initiation Grant from the University of Santiago de Compostela, co-financed by Banco Santander. The funders were not involved in the study design, collection, analysis, interpretation of data, the writing of this article or the decision to submit it for publication.

## Conflict of Interest

The authors declare that the research was conducted in the absence of any commercial or financial relationships that could be construed as a potential conflict of interest.

## Publisher's Note

All claims expressed in this article are solely those of the authors and do not necessarily represent those of their affiliated organizations, or those of the publisher, the editors and the reviewers. Any product that may be evaluated in this article, or claim that may be made by its manufacturer, is not guaranteed or endorsed by the publisher.
