# Supplementary material for: Cognitive Frailty: An Update
Source: Front Psychol. 2021 Dec 16;12:813398. doi: 10.3389/fpsyg.2021.813398 (PMC8717771; doi:10.3389/fpsyg.2021.813398)
Supplement: Supplementary file 1 [file Table_1.DOCX]

Table 1.

| Authors (year) | Study design (sample size) | Definition of cognitive frailty (CF) | Test(s) used to assess CF | Main outcomes |
| --- | --- | --- | --- | --- |
| Armstrong, Andrews, Gross, Varma, Xue and Carlson (2019) | Data from 607 participants of the Baltimore Experience Corps Trial. | CF as the end of the continuum of cognitive resilience with an elevated risk of cognitive impairment and dementia. | The Cognitive Frailty Index (CFI), a brief computerized Stroop game designed to assess trial-based learning under easy (naming colour of X’s) and challenging (naming colour of colour-words) conditions | The association between the Cognitive Frailty Index (CFI) and physical functioning demonstrates the interplay between CF and worsened objective mobility in a sociodemographically at-risk sample. Those participants with poorer physical function were also slower in the CFI and did not improve much from trial to trial. |
| Gallucci et al. (2020) | 46 participants in the intervention group and 161 in the control group, all outpatients with MCI attending a Cognitive Impairment Center in Treviso, Italy | CF defined as a multidimensional construct that identifies a status of heightened vulnerability to negative health-related events. | Comprehensive testing, including clinical, cognitive, behavioural, functional, physical and neuroimaging assessment. | CF status was likely to improve in the intervention group by more than three times the rate in the control group. The participants in the control group showed twice the risk of developing CF. Age, vascular state and neuropsychiatric symptoms were associated with worsening frailty status, while level of education was a protective factor. |
| He et al. (2020) | Cross-sectional study, with a total of 451 adults aged 65 years or older. | I.A.N.A - I.A.G.G definition. | Frailty status was determined using a frailty index constructed with 48 variables according to the cumulative deficits model. | Plasma TMAO levels were significantly higher in frail than in non-frail old adults. Linear association between the TMAO concentration and OR for physical and cognitive frailty. |
| Hsieh et al. (2018) | 2386 old adults selected from the Healthy Aging Longitudinal Study in Taiwan (HALST) study. | I.A.N.A - I.A.G.G definition, including reversible and potentially reversible subtypes. | Fried’s frailty phenotype, MMSE, self-reports about forgetting to take regular medication during the past year. | Old adults with SCD are more likely to be categorised as pre-frail or cognitively frail than those with normal cognition. Association between SCD and frailty in unintentional weight loss, slowness and low level of physical activity. |
| Kim et al. (2019) | Cross-sectional data from 1192 community-dwelling older people from Tokyo, Japan. | I.A.N.A. / I.A.G.G. definition. Operationally, cognitive frailty was defined as scoring ≥3 in the physical frailty criteria and having an MMSE score between 19 and 26 points, without dementia. | Mini-Mental State Examination, calf circumference, Timed Up and Go (TUG), usual walking speed, Council on Nutrition Appetite Questionnaire (CNAQ) and physical frailty criteria. | Age, chronic disease, more time required to complete the TUG and poorer nutritional status were significantly associated with CF and CF-related falls. Greater calf circumference and a good nutritional status have protective effects. |
| Kwan et al. (2020) | 33 older adults from 2 senior community centers in Hong Kong | I.A.N.A. / I.A.G.G. definition. | MoCA, Fried’s Frailty Index. | Brisk walking training was effective in improving cognitive function after 12 weeks in both conventional behaviour change intervention and mHealth intervention groups. In this last group, significant improvement was also observed in frailty reduction, walking time, step count, brisk walking time, peak cadence and moderate-to-vigorous physical activity time. |
| Liu et al. (2018) | 1298 older adults from the Lifestyle Interventions and Independence for Elders (LIFE) Study, in eight US field centers. | I.A.N.A./I.A.G.G. definition. | Modified Mini-Mental State Examination (3MSE) and Short Physical Performance Battery, Study of Osteoporotic Fractures (SOF) index and plasma IL-6 was measured at baseline. | A 24-month structured, moderate-intensity physical activity programme reduced the risk of worsening CF than a health education programme in sedentary older adults. The effect did not differ according to baseline IL-6 levels. |
| Niederstrasser et al. (2019) | 8780 older adults from the English Longitudinal Study of Ageing  (ELSA). | Calls to include a cognitive domain within frailty constructs. Evidence that cognitive decline and frailty share common pathologies. | Frailty index including self-reported health, disabilities, cognitive function, hearing, eyesight, depressive symptoms and ability to carry out activities of daily living. | Participants with no educational qualifications (vs. any educational qualification) showed a significant risk for early development of frailty and for frailty progression 12 years later. |
| Okura et al. (2019) | Cross-sectional data from 5.094 older adult residents in Kami Town, Japan. | Following I.A.N.A./I.A.G.G. definition, self-reported mobility decline (SR-MD) and self-reported-cognitive decline (SR-CD) is studied. | The Kihon Checklist (KCL), which includes self-reported items about physical strength, nutrition, eating, socialization, memory, mood and instrumental activities of daily living. Based on the total scores for SR-MD index and SR-CD index, participants were categorized into non-SR-cognitively frail, non-SR-MD & SR-CD, SR-MD & non-SR-CD, and SR-cognitively frail. | The impact of self-reported cognitive frailty on adverse health outcomes depends on age and sex. |
| Razjouyan et al. (2018) | 153 community-dwelling older adults who were able to walk 15 feet (4.5 m) independently, with or without aid. | Fried’s frailty phenotype criteria. | Five core clinical criteria underlying frailty: shrinking, exhaustion, inactivity, slowness and weakness. | Pendant sensor can identify stepping parameters, walking parameters and sedentary and moderate-to-vigorous activity and thus identify pre-frail old adults. No sleep parameters identified pre-frailty. |
| Razjouyan et al. (2020) | 163 community-living older adults ≥ 60 years of age without severe gait or balance disorders, of which 23 presented cognitive fragility. | I.A.N.A - I.A.G.G definition. | Fried’s frailty phenotype and Mini-Mental State Examination (MMSE). Remote monitoring using wearable sensors to determine daily physical activity and parameters. | Using a decision tree classifier, six independent sensor-derived parameters representing cumulative postures (duration of standing and walking at percentage over 24 h), activity behaviour (percentage of sedentary behaviour, percentage and total moderate-to-vigorous activity over 24 h), and locomotion (total number of steps over 48 h) distinguished older adults with cognitive frailty from age-matched older adults with a sensitivity of 0.93 and a specificity of 0.57. |
| Rezola-Pardo et al. (2019) | 188 older adults living in 9 long-term nursing homes in Gipuzkoa, Spain. | I.A.N.A./I.A.G.G definition. | Gait performance, functional assessment (SPPB, SFT, TUG, handgrip strength, Berg balance test, and objectively measured physical performance), cognitive performance (MoCA, Coding and Symbol Search from the WAIS-IV, TMT part A, Rey Auditory Verbal Learning Test) and emotional assessment (Jong Gierveld loneliness scale, QoL-AD). Frailty assessment included the Tilburg index, the Frailty index and the Rockwood scale. | Both single and dual-task multicomponent programmes were effective in improving gait performance and maintaining cognitive function. No group-by-time effects observed, except in the chair-stand test, which favoured the single physical exercise programme. |
| Rietman et al. (2019) | Cross-sectional study. 2220 participants from the Doetinchem Cohort Study | Accumulation of deficits, nor a multidimensional approach to studying frailty with little agreement between instruments. | Multidimensional approach to studying frailty. Physical frailty was defined according to the Frailty Phenotype and Tilburg Frailty Indicator. Cognitive performance was tested using Words Verbal Learning Test (VLT) (immediate and delayed recall), Stroop Color–Word Test, Word Fluency Test and Letter Digit Substitution Test. | Of the 17 biomarkers included, differential trajectories for three biomarkers were only observed in women: total cholesterol, gamma-glutamyltransferase and urea. |
| Rivan et al. (2020). | Of 815 older adults participating in the Malaysian Towards Useful Aging (TUA) study, a total of 282 participants without CF at baseline were successfully followed up. | I.A.N.A - I.A.G.G definition. | Fried frailty criteria, and Petersen criteria for MCI. | The 5 years’ cumulative incidence of cognitive frailty was 35.5%. Advanced age, depression, decreased processing speed, decreased functional mobility measured with TUG, low vitamin D intake and physical frailty were predictors of the incidence of cognitive frailty. |
| Romera-Liebana et al. (2018) | 352 community-dwelling cognitively frail or prefrail adults, aged older than 65 | Continuum from a pre-frail stage to mild impairment without a total loss of function and potential reversibility at early stages. | Short Physical Performance Battery, neurocognitive battery (including verbal memory, semantic and phonetic fluency, naming, phonological retrieval, visual recognition memory and verbal abstraction) and modified Fried’s criteria. | A physical, nutritional, neurocognitive and pharmacological multifaceted intervention was effective in improving functional and cognitive measures and in reducing the number of prescriptions both in the short term (3 months) and intermediate term (18 months). |
| Royal and Palmer (2019) | Longitudinal study. 2551 participants from the Texas Alzheimer’s Research and Care Consortium. | CF defined as the cause of age-specific functionally salient cognitive impairment (i.e. ‘senility’). | Cognitive performance tested with the Animal naming test, Boston Naming Test, CDR scale ‘Sum of Boxes’, Wechsler Logical Memory immediate recall, Wechsler Logical Memory delayed recall, MMSE; Subjective cognitive impairment and TMT- B. | Blood-based protein biomarkers related to CF. |
| Ruan et al. (2020) | Cross-sectional data of 5328 participants from a Shanghai study on health promotion for frail old. individuals. | I.A.N.A - I.A.G.G definition, including reversible and potentially reversible subtypes. | 5-item self-reported FRAIL scale, 3-item Rapid Cognitive Screen tools and a modified subjective cognitive decline questionnaire. | Respectively 19.86% and 6.30% of the participants exhibited reversible and potentially reversible CF.  Physically frail participants showed a high risk of MCI but a low risk of SCD, whereas participants with physical pre-frailty showed high risks of MCI and SCD. |
| Sargent et al. (2020) | Longitudinal study from the Aging in Chianti (InCHIANTI Study) with a representative sample of 1453 older adults from Tuscany, Italy. | CF measures included the number of frailty symptoms as defined in the cardiovascular health study (CHS). | MMSE as a test of general cognition and Trail Making Test, part A and B (TMT). | Model I tested prediction of genetic, protein and clinical markers of cognitive frailty using criteria from MMSE, while Model II used the TMT, Parts A and B. Both models developed were highly predictive. |
| Sugimoto et al. (2019) | Cross-sectional study, 233 patients of the Memory Clinic at the National Center for Geriatrics and Gerontology of Japan. | The PF based on the frailty phenotype proposed by Fried et al. in the Cardiovascular Health Study (CHS). CF was defined as simultaneous presence of pre-PF or PF and MCI. | MMSE, Frailty components (slowness, weakness, shrinking, exhaustion and low level of physical activity). | Association between CF and WMH in memory clinic outpatients. Cognitively frail participants had higher WMH volumes than cognitively normal counterparts. PF/cognitively normal, pre-PF/MCI, and PF/MCI groups had higher WMH volumes than the non-PF/cognitively normal group. |
| Tsutsumimoto et al. (2018) | Cross-sectional observational study with 10.885 community-dwelling older adults registered in the National Center for Geriatrics and Gerontology – Study of Geriatric Syndromes. | Simultaneous presence of both physical frailty (slow walking speed and/or muscle weakness) and cognitive impairment (at least 1.5 standard deviations below the threshold after adjusting for age and education) | Mini-Mental State Examination (MMSE), word list memory-I and word list memory-II, tablet versions of the Trail Making Test (TMT) and the Symbol Digit Substitution Test (SDST). | In addition to being associated with falls, CF is more closely associated with fall-related fractures than cognitive impairment or physical frailty alone. |
| Wan et al. (2020) | Cross-sectional design studying 26 older adults with CF and 26 matched healthy subjects | I.A.N.A - I.A.G.G definition | Cognitive functioning and physical frailty were assessed with the Montreal Cognitive Assessment (MoCA) scale (Fuzhou version) and the Chinese version of the Edmonton Frailty Scale (EFS). | Changes in subcortical nuclei in older adults with CF are related to cognitive decline and physical frailty |
| Wanaratna et al. (2019) | Cross-sectional study of 780 community-dwelling older patients with knee osteoarthritis in four representative cities of Thailand. | I.A.N.A - I.A.G.G definition, operationalized using the MiniCog (impaired MiniCog = 0–2) for evaluation of cognitive function and Fried’s phenotype for physical frailty. | Fried phenotype, Mini Mental State Examination (MMSE), Mini Nutritional Assessment Short, Barthel’s ADL, Patient Health Questionnaires, Global Physical Activity Questionnaire. | Prevalence of CF was 2.44%. Prevalence of CF and pre-frailty was high in knee OA aging, severe knee OA symptoms, malnutrition, and functional dependence were associated with CF. |
| Wongtrakulruang et al. (2020) | Convenience sampling of 195 patients from medical outpatient clinics of three hospitals in Bangkok. | I.A.N.A - I.A.G.G definition. Cognitive frailty was based on positive results in at least three of the Fried criteria and MCI. Cognitive pre-frailty was based on positive results for 1-2 items of the Fried criteria and MCI. | Modified Fried’s frailty phenotype, 5-item self-reported FRAIL scale, Thai Mental State Examination (TMSE) and Montreal Cognitive Assessment-Basic (MoCA-B). | The prevalence of cognitive pre-frailty was 14.4% and that of CF, 6.7%. Age ≥70 years and education at primary school or under were associated with higher risk of cognitive frailty/pre-frailty. Correlations between Fried’s criteria and the FRAIL scale were moderate to high. |
| Yoon et al. (2018) | 65 community-dwelling older adults living in Seoul, Korea. | I.A.N.A./I.A.G.G definition. | Cardiovascular Health Study (CHS) frailty phenotype criteria, MMSE, CERAD battery (memory, processing speed, cognitive flexibility, working memory, executive function), physical function (SPPB, TUG, gait speed), and muscle strength (grip strength, knee extension strength). | Interventions with high-speed resistance exercise training were effective in improving cognitive function (processing speed and executive function), physical function (gait speed, chair stand, balance and general performance in Timed Up and Go test), and muscle strength (grip strength, knee extension strength), although significant changes in frailty scores were not found. |
| Zhao et al. (2020). | Cross-sectional study with 3.242 rural old adults from Shandong, China. | I.A.N.A - I.A.G.G definition. | Frailty phenotype of Fried’s and the Mini Mental State Examination (MMSE). Falls were evaluated by the question “Have you ever fallen during the past 12 months?” Engagement in activity was measured on the basis of the China Health and Retirement Longitudinal Study. | Association between CF and falls in rural older adults. Prevalence of falls was 13.1% and prevalence of cognitive frailty was 6.6%. Engagement in activity mediated this association. |
| Zhou et al. (2018) | 32 community-dwelling older adults, aged 65 years or older. | Low physiological reserve and vulnerability to illness, with high risk of disability, institutionalization, and death. | Mini-Mental State Examination (MMSE) or Montreal Cognitive Assessment (MoCA), Instrumented Trail Making Test (TMT), and walking test.  TMT tasks included reaching five target circles, including the numbers 1-to-3 and letters A&B placed in random order, on the computer-screen by moving the ankle-joint while standing. The participants used a wearable sensor to quantify motor planning errors (MPE), an index that is based on ankle velocity pattern measurements during the TMT. | Differences between participants with and without cognitive impairment. Significant correlations between the MPE and MoCA test results, as well as between the MPE and dual task stride velocity results. |
